# Dynamic Spatiotemporal Shifts of Cerebral Microbleeds and Intracerebral Hemorrhage Over 11 Years in a Patient With Mixed Cerebral Small Vessel Disease Phenotype: A Case Report and Literature Review

**DOI:** 10.7759/cureus.104298

**Published:** 2026-02-26

**Authors:** Masahiro Hayashi, Hiroko Akamatsu, Katsuji Kobayashi

**Affiliations:** 1 Department of Neurology, Juuzen Hospital, Kanazawa, JPN; 2 Department of Psychiatry, Asanogawa Medical Corporation, Sakuragaoka Hospital, Kanazawa, JPN; 3 Department of Psychiatry, Awazu Neuropsychiatric Sanatorium, Komatsu, JPN

**Keywords:** cerebral microbleeds, cerebral small vessel disease, epileptic seizures, intracerebral hemorrhage, mixed cerebral small vessel disease

## Abstract

This report describes a patient with a mixed cerebral small vessel disease (CSVD) phenotype in whom approximately 11 years of longitudinal neuroimaging and clinical follow-up enabled an integrated assessment of (1) the temporal and spatial relationships between cerebral microbleeds (CMBs) and intracerebral hemorrhage (ICH), (2) the dynamic evolution of the mixed CMB distribution, and (3) the association between CMB progression and epileptic seizures.

From an early stage, CMBs were present in both lobar and deep regions. Over time, their distribution expanded from lobar to deep and subsequently to infratentorial regions, and the locations of symptomatic ICH shifted in parallel. In both lobar and deep regions, ICH occurred within areas showing new CMB formation, clustering, or morphological changes.

Notably, in the left parieto-occipital region, an ICH developed in very close spatial proximity to dynamically changing CMBs, strongly suggesting continuity between microvascular injury reflected by evolving CMBs and subsequent macrohemorrhage.

Epileptic seizures were not fully explained by single ICH events or cross-sectional CMB burden alone. Instead, seizures tended to occur during a phase of rapid emergence and increase of lobar CMBs, and no seizure recurrence was observed after the CMB count reached a plateau. These findings raise the possibility that dynamic CMB activity may reflect an active phase of cortical small vessel injury and may be associated with seizure susceptibility.

After stabilization of blood pressure and daily routines following institutionalization, new CMB formation decreased, recurrence of epileptic seizures and ICH events was suppressed, and basic activities of daily living were largely preserved. Although this is a single-case observation and cannot be generalized, long-term longitudinal imaging suggests that mixed CSVD phenotype may be better understood as a dynamic spectrum disorder. In addition to quantitative CMB burden, qualitative and temporal CMB dynamics may represent imaging markers of disease activity and may be temporally associated with ICH and epileptic seizures in selected patients.

## Introduction

Cerebral small vessel disease (CSVD) comprises a group of disorders caused by structural and functional impairment of small intracerebral vessels, including small arteries, arterioles, capillaries, and venules [1,2]. CSVD accounts for approximately 20-30% of all strokes [1] and is also associated with cognitive impairment and epileptic seizures in older adults [3,4]. Among its major pathological subtypes are hypertensive arteriolosclerosis (HTN-SVD), driven by vascular risk factors such as hypertension, and cerebral amyloid angiopathy (CAA), characterized by amyloid-β (Aβ) deposition in vessel walls [1,2,5].

Advances in neuroimaging have expanded the recognized phenotypic spectrum of CSVD beyond lacunes and white matter hyperintensities to include cerebral microbleeds (CMBs), enlarged perivascular spaces, and cortical superficial siderosis (cSS) [1,2,6]. CMBs appear as well-defined hypointense lesions on T2-weighted imaging or susceptibility-weighted imaging (SWI), and their number and spatial distribution have been linked to stroke, cognitive decline, and seizures [1,3,7].

Classically, HTN-SVD is associated with deep and infratentorial CMBs, whereas CAA is characterized by predominantly lobar CMBs. However, a mixed CMB distribution involving both lobar and deep regions, often described as a mixed CSVD phenotype or mixed-location CMB pattern, has increasingly been recognized [5]. Such a mixed CSVD phenotype may reflect either the coexistence of HTN-SVD and CAA or a hypertensive microangiopathy phenotype extending across vascular compartments, as suggested by amyloid positron emission tomography (PET) studies [8]. Nevertheless, the pathophysiology and dynamic evolution of mixed CSVD, particularly the temporal and spatial relationships between CMBs and intracerebral hemorrhage (ICH), remain insufficiently understood [5,9].

Here, we report a patient with a mixed CSVD phenotype who was followed in detail over 11 years (age 58.7-69.7 years). CMBs were present in both lobar and deep regions from the earliest observation. During long-term follow-up beginning with the first symptomatic ICH at age 58.7 years, the distribution of hemorrhagic lesions progressively extended from lobar to deep and later to infratentorial regions, with symptomatic ICH locations shifting in parallel. We describe the clinical course and longitudinal imaging findings and discuss the pathophysiology of mixed CSVD, the relationship between CMBs and ICH, and the potential link between dynamic CMB activity and epileptic seizures.

## Case presentation

Clinical course

The patient had been diagnosed with hypertension in his early 50s and received antihypertensive therapy, although blood pressure control remained insufficient. At age 58.7 years, intermittent headaches prompted brain imaging, which revealed a subacute ICH, leading to hospital admission. Neurological examination showed no focal deficits; however, executive dysfunction and attention impairment were noted, and the Mini-Mental State Examination (MMSE) score was 20 [10]. The ICH decreased with conservative management, and the patient was discharged home.

From age 58.7 to 66.6 years, seven symptomatic ICH events were identified. At age 59.7 years, during the second ICH event, the first epileptic seizure occurred. Seizures recurred thereafter, with a total of seven events until age 63.1 years, requiring long-term antiseizure medication. Several electroencephalograms (EEGs) performed after the initial seizure did not reveal definite epileptiform discharges; in the later period, generalized slowing was observed, predominantly in the 6-8 Hz range.

At age 63.1 years, the fourth ICH event coincided with the seventh seizure event. After this period, cognitive decline and gait disturbance became more evident, and the patient required institutional care due to reduced instrumental activities of daily living (IADL). The MMSE score at that time was 17. Following admission to a care facility, medication adherence, diet, and daily routines became stable, and blood pressure control improved. Between ages 64.3 and 66.6 years, three additional ICH events occurred; however, no seizures were observed during this period.

The primary antiseizure medication, perampanel, was gradually tapered and discontinued more than two years after the last seizure based on EEG findings, without seizure recurrence. Antihypertensive medications were also gradually reduced and discontinued under close blood pressure monitoring, without the subsequent elevation of blood pressure or increased diurnal variability. At age 66.6 years, the seventh ICH event occurred, again without prominent focal neurological deficits. Although the patient developed slowly progressive akinesia and postural instability requiring wheelchair use, basic activities of daily living (BADL) were largely preserved.

No symptomatic ICH recurrence was observed for more than three years after the seventh ICH event through the last follow-up at age 69.7 years. At age 69.1 years, the MMSE score had declined to 13, indicating further cognitive deterioration; however, BADL did not markedly deteriorate. Genetic testing performed during follow-up revealed an apolipoprotein E (APOE) ε3/ε3 genotype.

ICH and seizure events

**Figure 1:**
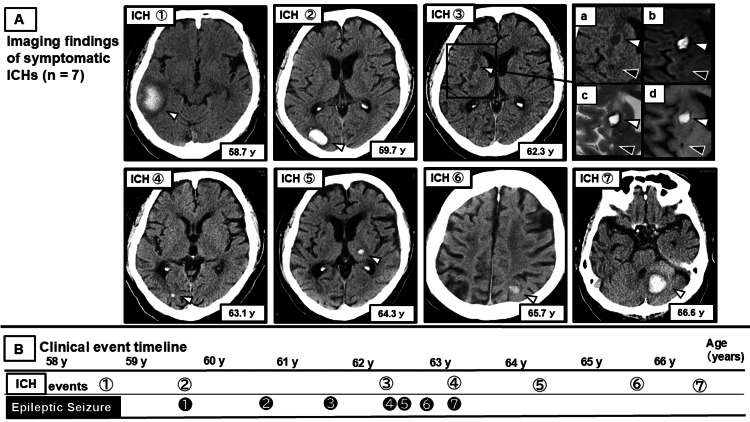
Longitudinal course of ICH and ESs Seven symptomatic ICH events (ICH 1-7) identified between the ages of 58.7 and 66.6 years, along with seven epileptic seizures (ES 1-7) occurring between the ages of 59.7 and 63.1 years, are shown in chronological order. ES 1 and ES 7 were classified as ASS, whereas ES 2-ES 6 were classified as LS. (A) Images of symptomatic ICH: CT images demonstrate seven symptomatic ICH events (ICH 1-7) identified during the observation period. White arrowheads indicate sites of ICH. The inset for ICH 3 (a: CT; b: DWI; c: T2WI; d: T1WI) shows magnified multimodal images of the region containing the hemorrhagic lesion. On CT, the hemorrhagic lesion corresponding to ICH appears as a faint hypodense area (white arrowhead), whereas it shows hyperintensity across the MRI sequences shown. In contrast, a lesion adjacent to the hemorrhage showing hypodensity on CT (black arrowhead) demonstrates iso- to mildly hypointense signal on DWI, hypointense signal on T1WI, and hyperintense signal on T2WI, findings consistent with a lacunar infarction. (B) Clinical event timeline: a timeline illustrates the temporal relationship between ICH events (ICH 1-7) and ESs (ES 1-7) based on the patient's age at onset. The numbering of ICH events corresponds to the CT images shown in image A. ICH: intracerebral hemorrhage; ESs: epileptic seizures; ASS: acute symptomatic seizure; LS: late seizure; CT: computed tomography; MRI: magnetic resonance imaging; DWI: diffusion-weighted imaging; T1WI: T1-weighted imaging; T2WI: T2-weighted imaging; y: years

Between ages 58.7 and 66.6 years, seven symptomatic ICH events were identified during longitudinal follow-up, primarily based on head CT (Figure 1A, 1B).

The first ICH occurred in the right temporal lobe and the second in the right occipital lobe. Subsequently, an ICH occurred in the right putamen, followed by a small recurrent lobar hemorrhage in the right occipital lobe. Later, an ICH developed in the contralateral deep region of the left thalamus. After a subsequent lobar ICH in the left parieto-occipital region, the final ICH occurred in the infratentorial region, involving the left cerebellum.

Seven seizure events were documented. The first seizure occurred at age 59.7 years during the acute phase of the right occipital lobar ICH and was classified as an acute symptomatic seizure (ASS). Recurrent events, including late seizures, occurred thereafter, whereas the final seizure occurred at age 63.1 during a lobar ICH event, again as ASS. Most seizures were characterized by leftward eye deviation and progression from left limb convulsions to generalized tonic-clonic seizures, suggesting a focal onset in the right hemisphere. The seven seizure events clustered within approximately 3.4 years after the initial seizure, with no recurrence thereafter. Detailed clinical characteristics and timing of each ICH and associated seizure events are summarized (Table 1).

**Table 1:** Temporal relationship between ICH and ESs The table summarizes a total of seven symptomatic ICH events identified between the ages of 58.7 and 66.6 years and seven ESs that occurred between the ages of 59.7 and 63.1 years. For each ICH episode, the hemorrhage location (lobar, deep, or infratentorial), the presence or absence of seizures, seizure type (ASS or LS), and the temporal relationship between ICH onset and seizure occurrence are shown. Detailed lesion characteristics, anatomical locations, and the age at onset for each ICH episode are illustrated in Figure 1A, while the chronological relationship between ICH and ESs is shown in Figure 1B. ICH: intracerebral hemorrhage; ESs: epileptic seizures; ASS: acute symptomatic seizure; LS: late seizure; FS: focal seizure; CT: computed tomography; MRI: magnetic resonance imaging; y: years

ICH no.	ICH type	Age at event (y)	ES	Relation to ICH	Figure ref
ICH 1	Lobar	58.7	N.A.	N.A.	Figure 1A, 1B (ICH 1)
ICH 2	Lobar	59.7	ASS	0 days	Figure 1A, 1B (ICH 2, ES 1)
N.A.	N.A.	60.8	FS	~12 months	Figure 1B (ES 2)
N.A.	N.A.	61.5	FS	~22 months	Figure 1B (ES 3)
ICH 3	Deep	62.3	FS	>1 week	Figure 1A, 1B (ICH 3, ES 4)
N.A.	N.A.	62.4	FS	~1 month	Figure 1B (ES 5)
N.A.	N.A.	62.8	FS	~6 months	Figure 1B (ES 6)
ICH 4	Lobar	63.1	ASS	0 days	Figure 1A, 1B (ICH 4, ES 7)
ICH 5	Deep	64.3	N.A.	N.A.	Figure 1A, 1B (ICH 5)
ICH 6	Lobar	65.7	N.A.	N.A.	Figure 1A, 1B (ICH 6)
ICH 7	Infratentorial	66.6	N.A.	N.A.	Figure 1A, 1B (ICH 7)

Longitudinal imaging evaluation of CMB progression and its relationship with ICH

Longitudinal SWI obtained over approximately 11 years was used to analyze the temporal and spatial relationships between CMB evolution, including emergence, distribution, and morphological changes, and subsequent ICH occurrence. Eight SWI examinations performed between ages 58.7 and 69.1 years were evaluated. CMBs were defined as round or ovoid hypointense lesions with clear margins measuring 2-10 mm in diameter. At each time point, the total number of CMBs was counted and categorized by location into lobar, deep, and infratentorial regions, and longitudinal morphological changes were assessed. CMBs were independently evaluated by two raters experienced in CMB assessment, with discrepancies resolved by consensus. All MRI examinations were performed using 3.0-T scanners; TP 1-TP 4 were acquired using a Philips Ingenia system, whereas TP 5-TP 8 were acquired using a Philips Ingenia Elition system following a scanner upgrade. Imaging protocols were kept as consistent as possible across examinations.

**Figure 2:**
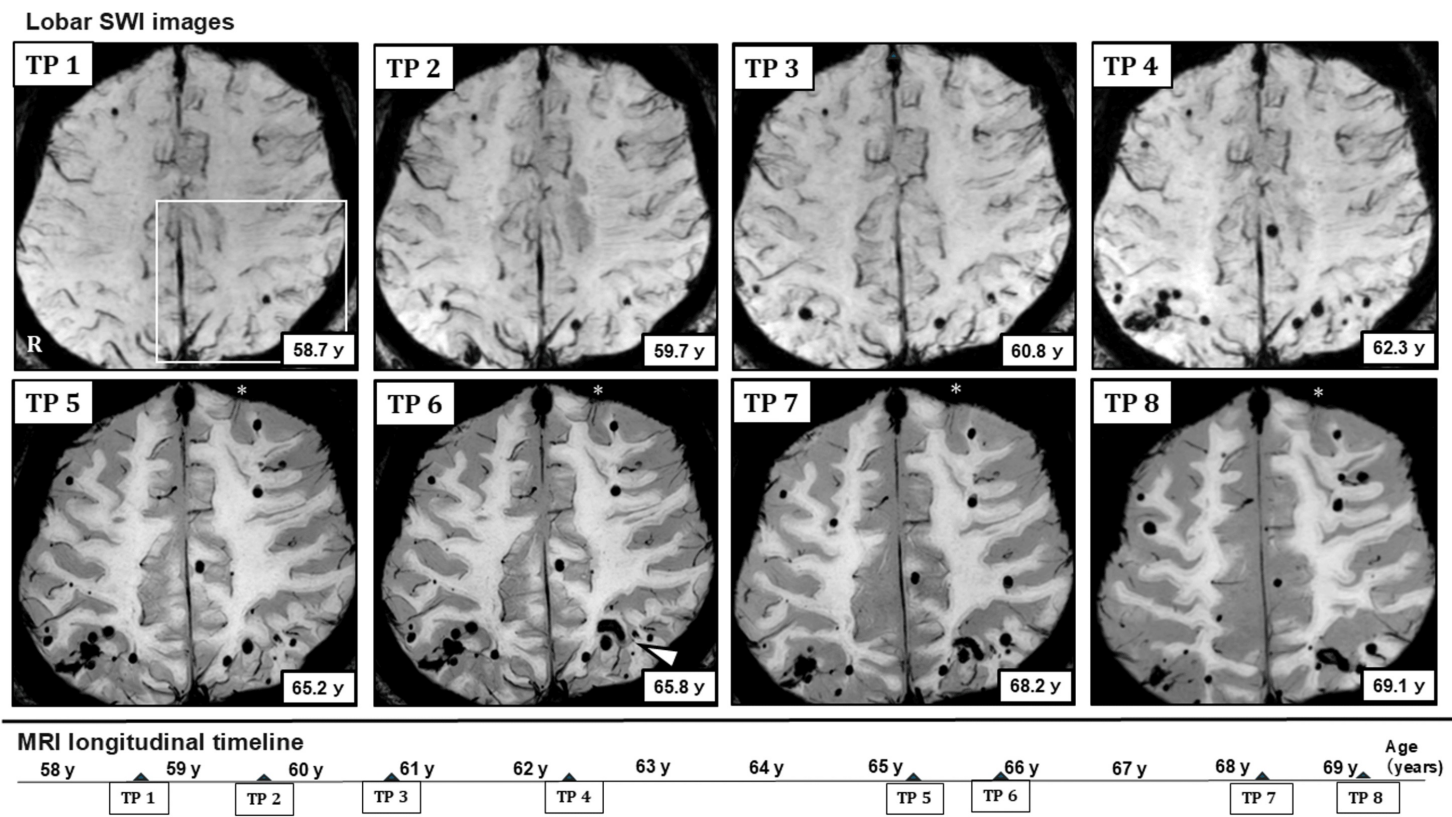
Longitudinal lobar SWI images showing the evolution of CMBs Longitudinal lobar SWI obtained at eight time points between the ages of 58.7 and 69.1 years demonstrates the spatial distribution, clustering, and morphological evolution of CMBs within the lobar regions. At the early stage, CMBs were predominantly observed in the bilateral parieto-occipital regions. During the middle stage, a rapid increase and clustering of CMBs occurred within the same regions. Over time, CMBs gradually extended from the parietal to the frontal lobes, while the formation of new CMBs and lesion enlargement became less prominent during the late stage. The white-outlined area at TP 1 indicates the left parieto-occipital region in which ICH (white arrowhead) subsequently developed following CMB clustering. In addition, cSS (asterisks) was observed in the left frontal lobe from the middle stage onward; however, these linear hypointense signals gradually became indistinct during the later phase. The patient's age at each imaging time point (TP 1-TP 8) is shown within the figure, and the timing of the eight SWI acquisitions is summarized chronologically in the lower panel. TP: time point; SWI: susceptibility-weighted imaging; CMBs: cerebral microbleeds; ICH: intracerebral hemorrhage; cSS: cortical superficial siderosis

**Figure 3:**
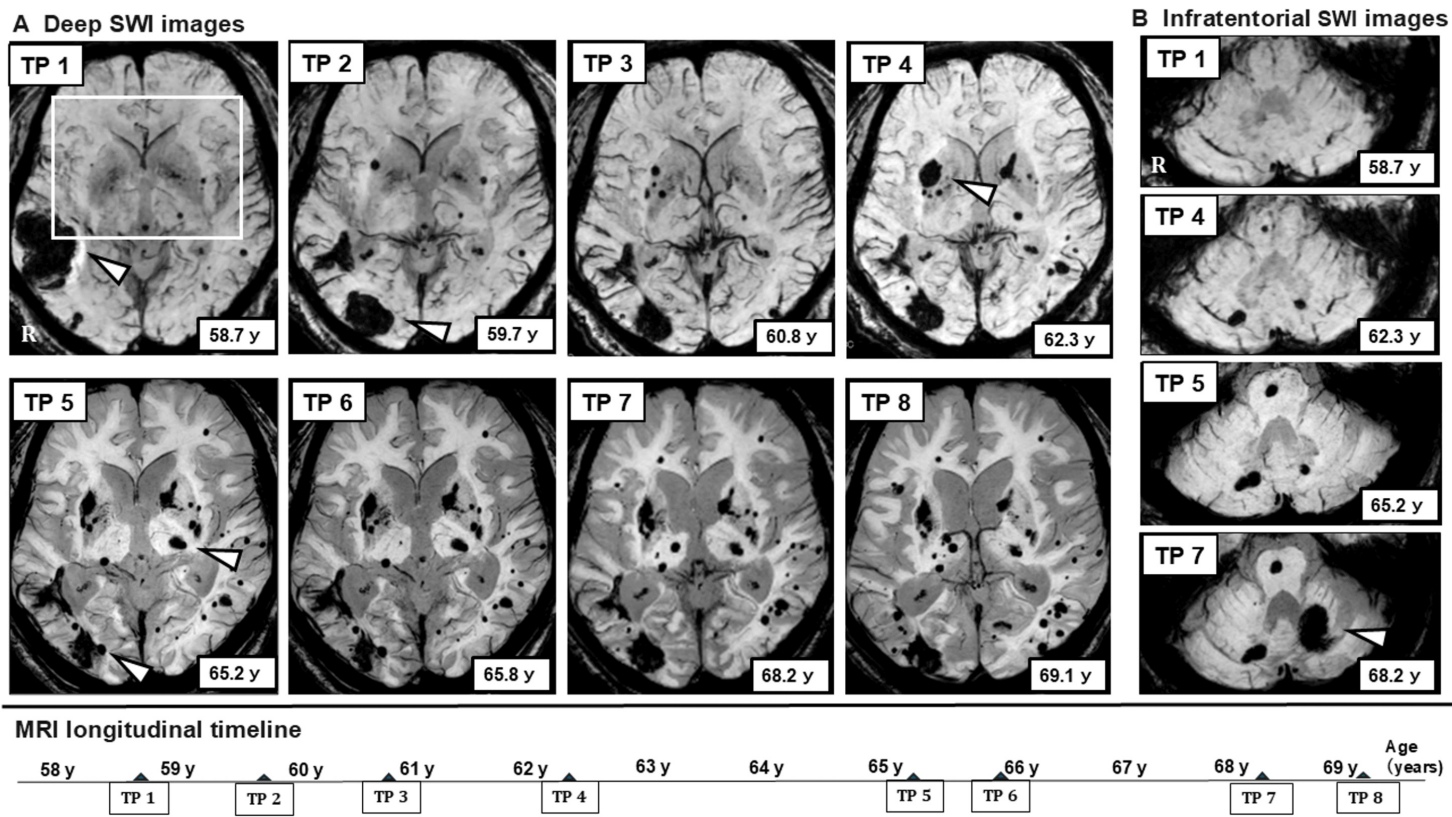
Longitudinal SWI changes in deep and infratentorial regions (A) Deep SWI images: longitudinal SWI focusing on the deep brain regions demonstrates temporal changes in CMBs across eight time points (TP 1-TP 8). At the early stage, new formation, clustering, and enlargement of CMBs were observed predominantly around the right putamen. Subsequently, an ICH developed within this region. After the occurrence of ICH, new CMBs emerged in the contralateral left putamen, and enlargement of a pre-existing CMB was observed in the left thalamus. Thereafter, an ICH developed in the left thalamus, followed by the appearance and increase of CMBs in the left putamen and surrounding areas, as well as new CMB formation in the contralateral right thalamus. White arrowheads indicate five sites of symptomatic ICH identified on SWI. The white-outlined area at TP 1 represents the bilateral putaminal and thalamic regions in which progressive CMB accumulation and subsequent ICH development were observed (see Figure 6). (B) Infratentorial SWI images: serial SWI images of the infratentorial region obtained at selected time points (TP 1, TP 4, TP 5, and TP 7) demonstrate the emergence and progression of CMBs. Following the initial appearance of CMBs in the right basis pontis and right cerebellar hemisphere, these lesions showed a tendency toward gradual enlargement. In contrast, the CMB located in the left cerebellar hemisphere, where a subsequent ICH eventually developed (white arrowhead), did not exhibit any clear enlargement prior to the hemorrhagic event. SWI: susceptibility-weighted imaging; TP: time point; CMBs: cerebral microbleeds; ICH: intracerebral hemorrhage

At the time of the first symptomatic ICH, SWI already demonstrated CMBs in both lobar and deep regions. Over time, CMB counts increased and the distribution expanded. In the lobar regions, new CMB formation and clustering developed predominantly in bilateral parieto-occipital areas, later extending toward parietal and frontal lobes. In the left frontal lobe, cSS appeared during the mid-phase; subsequently, the characteristic two-line hypointensity gradually became less conspicuous. In the deep region, CMB clustering developed around the right putamen, followed by ICH occurrence. Thereafter, infratentorial CMBs emerged, and the seventh symptomatic ICH later occurred in the left cerebellar hemisphere (Figure 2 and Figure 3A, 3B).

**Figure 4:**
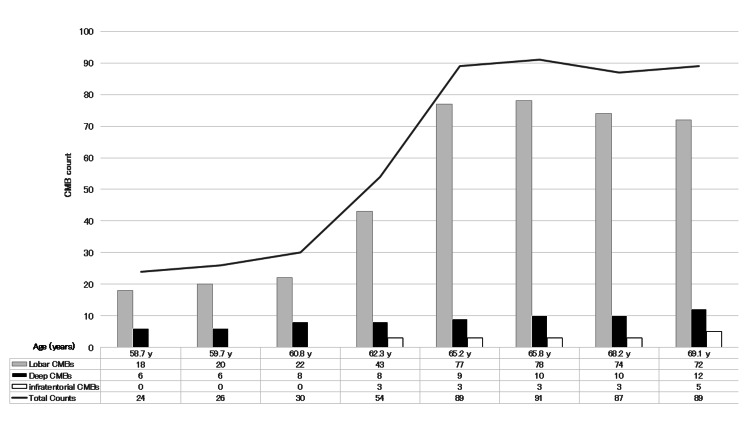
Longitudinal changes in the total number and distribution of CMBs The total number and distribution of CMBs (lobar, deep, and infratentorial) assessed on whole-brain SWI at each MRI time point (TP 1-TP 8) over the observation period are shown. CMBs: cerebral microbleeds; SWI: susceptibility-weighted imaging; TP: time point

Across the entire course, lobar CMBs accounted for approximately 70-80% of all CMBs. A rapid increase in lobar CMBs during the mid-phase was associated with a marked increase in the total CMB count. Subsequently, the formation of new lobar CMBs decreased, and the total count reached a plateau. In contrast, deep and infratentorial CMBs showed a gradual increase over time (Figure 4).

**Figure 5:**
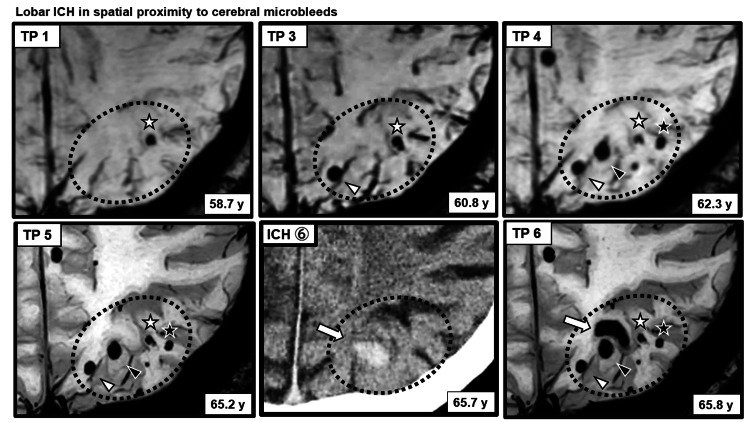
ICH arising in close spatial proximity to CMBs in a lobar region New formation and clustering of CMBs in the left parieto-occipital region (outlined by a dotted ellipse) and a subsequent ICH arising in close proximity are shown. Among the two CMBs located closest to the hemorrhagic focus, dynamic morphological changes were observed prior to ICH onset: one CMB showed enlargement (black arrowhead), whereas the other exhibited irregular deformation with a reduction in size (white asterisk). In contrast, two more peripheral CMBs (white arrowhead and black asterisk) did not demonstrate apparent size changes. The location of the corresponding ICH was confirmed on CT images shown in Figure 1. CMBs: cerebral microbleeds; ICH: intracerebral hemorrhage; SWI: susceptibility-weighted imaging; CT: computed tomography

Consistent with these quantitative trends, in lobar regions, ICH was observed adjacent to areas where new CMB formation or clustering was present (Figure 5).

**Figure 6:**
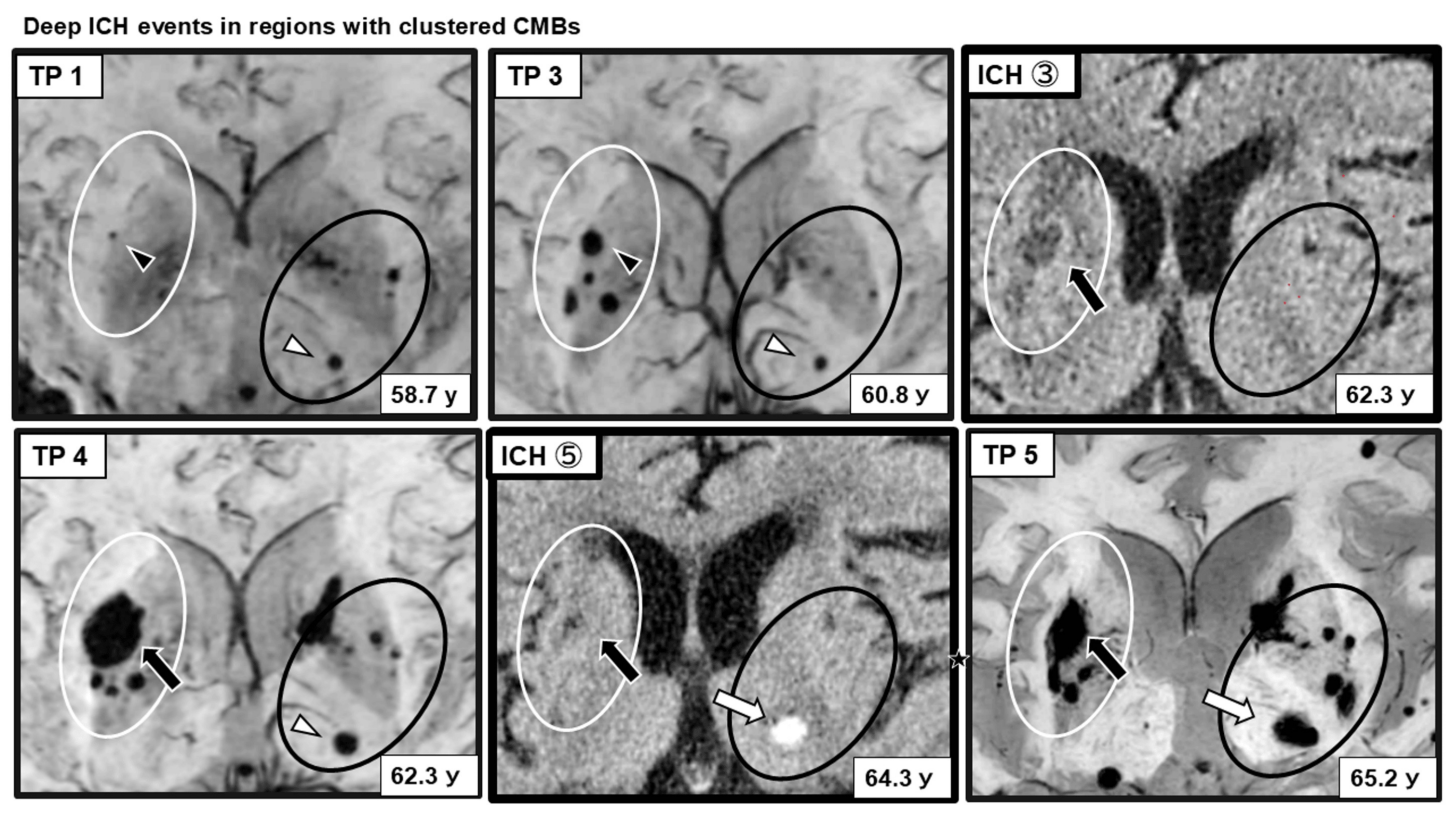
Deep ICH within regions containing multiple CMBs Representative SWI images demonstrating the clustering and progression of deep CMBs preceding ICH within affected deep regions. The timing and location of each ICH event correspond to those depicted in Figure 1. The white ellipse delineates the right putaminal region and its surrounding area. Within this region, the oldest CMB (black arrowhead) gradually enlarged over time, followed by the emergence and clustering of new CMBs, after which an ICH developed (black arrow). In contrast, the black ellipse indicates the region encompassing the left thalamus and left putamen. In this area, a pre-existing CMB in the left thalamus (white arrowhead) was present from an early stage and subsequently enlarged, after which an ICH occurred in the left thalamus (white arrowhead). In both deep regions, new CMBs emerged after ICH occurrence not only in the perilesional area but also in the contralateral hemisphere. SWI: susceptibility-weighted imaging; CMBs: cerebral microbleeds; ICH: intracerebral hemorrhage

In the deep regions, CMBs initially clustered in and around the right putamen; the oldest CMB also enlarged during the interval leading up to ICH occurrence within this cluster. After the hemorrhage, new CMBs appeared in the contralateral left putamen. In the left thalamus, enlargement of a pre-existing CMB was observed, followed by ICH. After the left thalamic ICH, new CMBs developed in the ipsilateral left putamen and contralateral right thalamus, gradually increasing over time. In the infratentorial region, CMBs emerged in the left pontine base and bilateral cerebellar hemispheres; enlargement of a right-sided CMB was observed, whereas the left cerebellar CMB did not demonstrate clear morphological change prior to subsequent ICH occurrence in the left cerebellar hemisphere (Figure 6).

## Discussion

In this report, the term mixed CSVD is used descriptively to denote a CSVD phenotype characterized by a mixed-location CMB distribution and longitudinal shifts in hemorrhagic manifestations, without implying a distinct or universally established disease entity.

Relationship between CMBs and ICH

In this patient, seven symptomatic ICH events were identified over approximately 11 years of longitudinal imaging follow-up, accompanied by dynamic changes in CMB number, distribution, and morphology. At the time of the first ICH, CMBs were already present in both lobar and deep regions, indicating a mixed-location CMB pattern and supporting a mixed CSVD phenotype [5,9]. Over time, CMBs extended to infratentorial regions.

The total number of CMBs increased rapidly during the early phase, reaching approximately 90 lesions and subsequently entering a plateau phase. Throughout the disease course, lobar CMBs accounted for approximately 70-80% of all CMBs. Previous studies have shown that higher CMB burden in mixed CSVD is associated with increased risk of ICH occurrence and recurrence [2,9]. In this case, the presence of numerous CMBs from an early stage suggests that quantitative CMB burden may have contributed to hemorrhagic vulnerability.

Importantly, prior studies have suggested that spatial relationships between CMBs and ICH differ by region [11]. In lobar areas, particularly in the left parieto-occipital region, ICH occurred adjacent to areas of newly formed and clustered CMBs. Because SWI and CT could be compared longitudinally at similar anatomical levels, spatial proximity between CMBs and subsequent ICH could be evaluated with relatively high confidence. In contrast, in deep regions such as the right putamen, CMBs and ICH overlapped spatially, making it difficult to identify the precise bleeding source on imaging alone. Similar limitations applied to ICHs in the left thalamus and left cerebellar hemisphere.

Although some reports suggest that CMBs themselves may serve as direct bleeding sources based on positional relationships [12], pathological studies indicate that CMBs represent chronic lesions following vessel rupture [13]. After vessel rupture, iron handling by microglia and astrocytes may persist, leading to ongoing iron-mediated toxicity and inflammatory responses that could secondarily damage surrounding tissue and adjacent vessels [11,12]. Therefore, in this case, fragile vessels adjacent to pre-existing CMBs, rather than the CMBs themselves, were more likely to serve as the source of subsequent ICH.

In addition to CMB clustering, this case demonstrated notable longitudinal CMB morphological changes preceding ICH. In the right putamen, ICH occurred after the clustering and enlargement of the oldest CMB. In the left thalamus, ICH followed the enlargement of a pre-existing CMB without marked clustering. In the left parieto-occipital region, two CMBs closest to the hemorrhagic focus exhibited dynamic changes, one shrinking with irregular contour changes and the other enlarging, before ICH onset. SWI depiction of CMBs reflects the blooming effect of paramagnetic iron within hemosiderin deposits [12,13], and changes in CMB size or morphology may reflect alterations in local iron distribution or concentration. Previous reports have documented the longitudinal enlargement or shrinkage of CMBs and ICH occurrence following CMB morphological change [14,15]. These spatial and temporal relationships between dynamically changing CMBs and subsequent ICH should be interpreted as associative and hypothesis-generating while acknowledging the difficulty in distinguishing true biological evolution from technical or methodological variation in longitudinal SWI assessment, as well as the possibility of minor or repeated microhemorrhagic events as an alternative explanation.

Furthermore, this case demonstrated CMB emergence or progression in regions remote from the primary hemorrhagic site, including contralateral deep structures and infratentorial regions. CSVD is considered a diffuse disorder involving global small vessel vulnerability and impaired cerebral autoregulation [16]. Hemodynamic stress superimposed on this global vulnerability may further contribute to hemorrhagic events in geographically distant vulnerable vessels [16]. In this patient, CMBs appearing in the contralateral hemisphere to deep ICH and those detected in the infratentorial region in temporal association with right putaminal ICH may reflect global vascular stress rather than a purely local hemorrhagic mechanism.

Taken together, the relationship between CMBs and ICH in this case appears to involve at least two interacting mechanisms: (1) local vascular fragility in the vicinity of CMBs and (2) global CSVD-related vascular vulnerability affecting the entire brain.

The interplay of these mechanisms across lobar, deep, and infratentorial regions may result in heterogeneous hemorrhagic phenotypes, including CMB emergence, clustering, morphological change, and overt ICH.

Pathophysiology of mixed CSVD phenotypes

CSVD is classically divided into HTN-SVD, associated with deep and infratentorial hemorrhagic lesions, and CAA, characterized by lobar-predominant hemorrhagic pathology [1,2,5]. Mixed CSVD phenotypes, defined by the presence of CMBs in both lobar and deep compartments, have gained increasing attention in recent years [5].

Compared with isolated HTN-SVD or CAA, mixed CSVD phenotypes have been reported to be more prevalent and to exhibit a higher total CMB burden, particularly with a high proportion of lobar CMBs [5,9]. In the present case, CMBs were present in both lobar and deep regions from the earliest observation, reached a maximum of approximately 90 lesions, and consistently showed lobar predominance, supporting a mixed CSVD phenotype.

The pathophysiology of mixed CSVD phenotypes remains debated. Proposed mechanisms include the coexistence of HTN-SVD and CAA or a severe hypertensive arteriopathy phenotype alone [5,9]. However, rather than rigid separation, a growing body of evidence supports viewing CSVD as a continuous spectrum influenced by aging, vascular risk factors, and amyloid-related pathology [17].

Experimental studies have demonstrated that hypertension-induced vascular narrowing and hypoperfusion can promote Aβ deposition in the vessel media [18]. Clinically, blood pressure lowering has been reported to reduce ICH recurrence more effectively in patients with CAA than in those with pure HTN-SVD [19].

In this patient, hemorrhagic lesions initially showed lobar predominance, which might have led to a diagnosis of CAA on cross-sectional assessment. However, long-term longitudinal follow-up revealed a gradual shift of hemorrhagic pathology from lobar to deep and eventually infratentorial regions. This temporal evolution suggests that mixed CSVD phenotypes are not fixed entities but rather dynamic conditions in which the dominant pathological expression may change over time depending on the relative contributions of aging, hypertension, amyloid burden, and genetic background. The APOE ε3/ε3 genotype in this patient may have modulated amyloid-related vascular pathology, further influencing this dynamic balance.

Accordingly, this case underscores that disease classification based on a single time point may be misleading and highlights the importance of longitudinal observation for the accurate characterization of mixed CSVD phenotypes as a dynamic spectrum disorder.

CMBs, ICH, and epileptic seizures

Post-stroke seizures occur in approximately 7% of patients and are particularly common after cortical ICH [4]. Seizures are typically classified as early seizures occurring within one week of stroke onset or late seizures occurring thereafter [4,7].

In this patient, seven seizure events were documented and classified into two ASS and five late seizures. Reported intervals for late seizures after ICH range from three to 23 months (mean approximately nine months) [7], and the seizure intervals observed in this case (maximum 22 months; mean 8.25 months) were broadly consistent with prior reports.

However, seizure occurrence could not be fully explained by single ICH events or cross-sectional CMB burden alone. Instead, seizures clustered during a period of rapid increase in lobar CMBs. After the CMB count reached a plateau, seizure recurrence was not observed. These findings suggest that longitudinal imaging assessment may provide insights beyond cross-sectional evaluation in identifying periods of increased seizure susceptibility.

Clinical management and prognostic implications

Hypertension is a major modifiable risk factor for CSVD, and strict blood pressure control remains central to clinical management. Lowering systolic blood pressure below 130 mmHg significantly reduces the risk of ICH recurrence [18].

In this patient, stabilization of blood pressure and daily routines following institutionalization was temporally associated with a reduction in new CMB formation, seizure resolution, and the suppression of further ICH events. Although causal inference cannot be established from a single case, these observations raise the possibility that stabilization of vascular stress may attenuate periods of heightened microvascular instability.

Despite these observations, this report is limited by its single-case design. Therefore, the associations described should be regarded as hypothesis-generating.

## Conclusions

This long-term longitudinal case illustrates that the mixed CSVD phenotype may be better understood as a dynamic spectrum disorder rather than a fixed pathological entity. In addition to quantitative CMB burden, temporal dynamics of CMB emergence, clustering, and morphological change may reflect disease activity and may be temporally associated with ICH and epileptic seizures. Longitudinal imaging assessment may therefore provide insights beyond cross-sectional evaluation in selected patients with CSVD.
